# Crosstalk Between Autophagy and Cerebral Ischemia

**DOI:** 10.3389/fnins.2018.01022

**Published:** 2019-01-14

**Authors:** Yulin Sun, Yuanhan Zhu, Xiaojun Zhong, Xinle Chen, Jun Wang, Guozheng Ying

**Affiliations:** Department of Neurosurgery, Zhejiang Rongjun Hospital, Jiaxing, China

**Keywords:** autophagy, cerebral ischemia, mechanism, apoptosis, target

## Abstract

With the use of advanced electron microscopy and molecular biology tools, several studies have shown that autophagy is involved in the development of ischemic stroke. A series of molecular mechanisms are involved in the regulation of autophagy. In this work, the possible molecular mechanisms involved in autophagy during ischemic stroke were reviewed and new potential targets for the study and treatment of ischemic stroke were provided.

## Introduction

The incidence of ischemic stroke has increased in recent years, accounting for 60–80% of all strokes. Generally, hypoxic ischemic encephalopathy and acute cerebrovascular accidents cause insufficient blood flow to the brain tissue, which in turn leads to brain cell metabolic disorders, leading to brain cell death, and irreversible damage to tissues. Thrombolytic therapy is a clinically effective treatment, but its limited time window and the associated high rate of recurrence limit its clinical application. Therefore, there is an extremely urgent need to find new and effective therapeutic targets and drugs for ischemic stroke.

Autophagy is a phagocytic degradation process of foreign bodies, damaged or aging organelles in the cytoplasm by autophagy lysosomal system. It belongs to non-caspase-dependent programmed death. Due to the transport and properties of active proteins after autophagy mitosis, neuronal survival is highly dependent on autophagy under physiological conditions. However, recent studies have shown that (Liu et al., 2018; Wang P. et al., 2018) after ischemic stroke, autophagy is activated and may be involved in the development of ischemic stroke. A series of molecular mechanisms are involved in the regulation of autophagy. This review focuses on the role of autophagy in ischemic stroke and its possible molecular mechanisms.

## Overview of Autophagy

Autophagy is derived from a Greek words meaning “phagy yourself.” It is a highly conserved cell behavior, mainly involved in the circulation as well as reuse of macromolecular substances in cells. It is also involved in the removal of damaged organelles, and plays an important role in maintaining the homeostasis of the intracellular environment.

Autophagy can be induced by changes in the internal conditions of the cell, such as organelles and cytoplasm accumulation or damage, or the cells are stimulated by external conditions, such as hunger, high temperature, hypoxia, and hormone stimulation (Doherty and Baehrecke, 2018). Mammalian autophagy is often divided into three types: macroautophagy, microautophagy, and chaperon mediated autophagy (CMA). In general terms, “autophagy” refers to large autophagy, which is responsible for the degradation of intracellular stable and persistent proteins to produce amino acids to maintain cell survival in the absence of nutrients. Microautophagy is a depression of the lysosomal membrane, direct phagocytosis of the cytoplasm, organelles, or nucleus to form autophagosomes, which are then degraded by lysosomal enzymes. The chaperone-mediated autophagy is selective, for instance, chaperone HSC70 recognizes a soluble cytosolic protein substrate with a KFERQ sequence and finally degrades the protein substrate with the KFERQ sequence. Its main role in the central nervous system is macrophagy and molecular chaperone-mediated autophagy (Nikoletopoulou et al., 2015).

## Relationship Between Autophagy and Ischemic Stroke

Mitochondrial dysfunction, acidosis, oxidative stress, calcium overload, excitotoxicity, and inflammatory response are involved in the development of cerebral ischemia-reperfusion injury (Halestrap, 2006), and leads to the accumulation of foreign bodies in the brain tissue to varying degrees. An increase in damaged cells may in turn induce the occurrence of autophagy. Nitatori et al. (1995) observed a significant increase in cathepsin B immunopositive lysosomes and an increase in autophagic phagocytosis using transmission of transient after cerebral ischemia in gerbils. This is the first time that autophagy was found to be activated in cerebral ischemia. Subsequently, the autophagosome structure was observed by transmission electron microscopy, and autophagy was confirmed to be involved in cerebral ischemia-reperfusion (I/R) (Kuma et al., 2004; Rami and Kogel, 2008; Li et al., 2018). Some researchers have further used pharmacological tools or autophagy-related knockout mice to study autophagy induction or inhibition, and verified the biological significance of functional autophagy in stroke (Li et al., 2018). The above evidence indicates that autophagy is involved in the development of stroke.

Recent studies have shown that (Morselli et al., 2008) acute and severe ischemia may cause “excessive autophagy,” thereby promoting cell death and damage. However, chronic and mild hypoxic state trigger “moderate autophagy,” thereby protecting cells by removing damaged tissues and proteins. It can be seen that during the development of cerebral ischemia, autophagy is a “double-edged sword.” However, regardless of the role of autophagy in ischemic stroke, a series of signaling pathways are required to complete the process involved.

## Possible Molecular Mechanisms of Autophagy Involved in Ischemic Stroke

### mTOR Signaling Pathway-Mediated Autophagy

Autolysosome reproduce (ALR) is a mammalian autophagy that extends into a tubular structure and separates the original lysosome, which further matures into a new lysosome. This process requires the activation of the mammalian target of rapamycin (mTOR). mTOR is a serine/threonine protein kinase, which includes mTORC1 (rapamycin sensitive) and mTORC2 (rapamycin under sensitive), where mTORC1 is the major regulatory target. Cellular responses to hypoxia and inflammation in mammals are signaled by the mTOR pathway, including induction of autophagy and cell survival (Sciarretta et al., 2018), where mTORC1 negatively regulates autophagy. When encountering oxygen sugar deprivation or using rapamycin, the kinase activity of mTORC1 is inhibited, thereby promoting autophagy. Hei et al. (2017) found that ischemic stroke can induce autophagy by inhibiting mTOR, and can alleviate the degree of cerebral ischemia in rats with acute hyperglycemia-induced cerebral ischemic injury, which may explain the conclusion that “moderate autophagy” may have a protective effect on the “slow and mild” ischemic brain damage.

The phosphoinositide 3-kinase (PI3K) protein family is involved in the regulation of various cellular functions such as cell proliferation, differentiation, apoptosis, and glucose transport. PI3K is an intracellular phosphatidylinositol kinase. The specificity of structure and substrate is divided into three types: I, II, and III. Among them, type III PI3K (Vps34) can form a complex with becline-1 to participate in the formation of autophagy. At the same time, it catalyzes the phosphorylation of phosphatidylinositol at D3 position to produce 3-phosphophosphatidylinositol, which recruits the “-FYVE-” or “-PX-” motif in the cytoplasm of the cell. This protein is used to form autophagosome membranes. Therefore, the formation of autophagosomes depends on the action of type III PI3K (Vps34). Akt is a major downstream effector of PI3K. Akt phosphorylates TSC1/2 (tuberous sclerosis complex), preventing its negative regulation of Rab (Ras homology, enriched in brain) and further activating Rheb enrichment and mTORC1. According to previous studies, after 3 h of ischemic stroke, the expression of protein kinase PI3K/Akt was significantly reduced; after 12 h, high levels of nerve growth factor (NGF) inhibited cystylation by activating the protein kinase PI3K/Akt signaling pathway, thereby reducing damage to the ischemic brain tissue. Thus, the PI3K/Akt signaling pathway is involved in the regulation of acute neurological damage during stroke (Shioda et al., 2009; Hong et al., 2014; Xu et al., 2018).

Previous study have shown that the selective autophagy inhibitor 3-methyladenine (3-MA) can prevent cerebral ischemia through the PI3K pathway in a time-dependent manner (Yu et al., 2017). Huang et al. (2018) found that curcumin can attenuate autophagy in nerve cells by activating the PI3K/Akt-mTOR pathway, thereby attenuating cerebral ischemia-reperfusion injury in adult rats. However, in the neonatal rat hypoxia model, after treatment with the mTOR inhibitor rapamycin, phosphorylation of p70S6K downstream of mTOR can be inhibited by activating the PI3K/Akt pathway, thereby inducing autophagy and exerting neuroprotection. On the other hand, 3-MA reduces the expression of the autophagy-related protein beclin1 and abolishes the neuroprotective effect of rapamycin (Carloni et al., 2010). The above evidence suggests that the PI3K/Akt-mTOR signaling pathway may be a new target for stroke. However, based on different ischemic animal models, the regulatory effects of PI3K/Akt on mTOR signaling and its effects on autophagy are inconsistent, and the corresponding mechanisms need to be further explored.

AMP-dependent protein kinase and autophagy play an important role in ischemic tolerance induced by cortical spreading depression (CSD), AMPK-mediated autophagy may represent a new target for stroke (Ronnett et al., 2009). The AMPK signaling pathway is an important pathway for enhancing autophagy in cell starvation. When the energy supply in the brain is reduced, the ATP/AMP ratio decreases, and AMPK is activated, which inhibits the downstream mTOR activity and activates autophagy to increase energy production (Dai et al., 2017; Wang J.F. et al., 2018; Zhang and Miao, 2018). Activated AMPK can inhibit mTORC1 in two ways: one is by regulating autophagy through TSC2 and mTOR regulation-related proteins; the other is by inducing autophagy by regulating the AMPK-mTOR pathway. AMPK-mediated autophagy contributes to the neuroprotection of ischemic preconditioning, suggesting that AMPK can be used as a target for the prevention and treatment of ischemic stroke (Liu H. et al., 2016).

In addition, Li et al. (2013) found that knocking out the p50 (NF-κB) gene during cerebral ischemia inhibited the Akt-mTOR pathway and enhanced autophagy, which in turn induced autophagic cell death. Cytoplasmic p53 can directly inhibit the formation of autophagosomes, while activated p53 translocates to the nucleus to promote AMPKβ expression, and transactivates sestrin-1, 2, and finally inhibits downstream mTOR activity to induce autophagy (Morselli et al., 2008). Brain ischemia/reperfusion can induce p53-dependent nuclear factor NF-κB expression while damage-regulated autophagy modulator (DRAM) is a positive regulator of p53-dependent autophagy. During the ischemia/reperfusion process, DRAM-mediated NF-κB/p53 signaling pathway is involved in apoptosis and autophagic cell death. Autophagy and apoptosis mechanisms can also participate in programmed cell death by regulating the p53 pathway (Cui et al., 2013). This suggests that the NF-κB-p53 signaling molecule is ultimately mediated by autophagy via mTOR, which may also serve as a potential target for stroke.

### MAPK Signaling Pathway-Mediated Autophagy

Mitogen activated protein kinase (MAPK) is composed of p38, extracellular regulated protein kinases (ERK), and c-Jun N-terminal kinase (JNK). Activation of p38 MAPK signaling pathway in early ischemic stroke promotes Elk1, CHOP10, LEF2C, and protein kinase MAPKK2/3 to maintain neuronal survival and exert anti-inflammatory and anti-apoptotic effects. In the late stage, p38 MAPK is over-activated, which may promote the expression of target genes by activating transcription factors and proteins such as caspase, etc., leading to neuronal apoptosis (Ferrer et al., 2003; Li et al., 2015; Song et al., 2016). Therefore, different interventions targeted at p38 MAPK signaling molecules should be administered at different periods of ischemic stroke. Related studies have confirmed the hypothesis mentioned above, and shown that numerous drugs that enhance autophagy by activating ERK, inhibiting JNK, and p38 MAPK, are beneficial for the treatment of ischemic stroke (Jiang et al., 2014; Vercelli et al., 2015; Wang et al., 2015). The Akt/Smads signaling pathway negatively regulates autophagy in PC12 cells induced by oxygen glucose deprivation (ODD) by inhibiting JNK and p38 MAPK molecules (Xue et al., 2016). p38 inhibitors promote cell survival signaling pathways (such as ERK), attenuate mitochondrial fragmentation caused by ischemia or mitochondrial autophagy, thereby reducing the volume of cerebral infarction after ischemia and protecting nerve function (Han et al., 2015). The above evidence suggests that ERK, JNK, and p38 MAPK mediate the molecular process of autophagy in ischemic stroke, in which ERK activates as well as inhibits autophagy, whereas JNK and p38MAPK produce opposite effects.

### HIF-1α Signaling Pathway-Mediated Autophagy

Molecular genetic studies have shown that the activity of hypoxia-inducible factor (HIF-1α) is closely related to ischemia-induced neuronal death. In the early stage of acute stroke, HIF-1α/HIF-2α double knockout in mice showed decreased expression of the anti-survival factors Bnip3, Bnip3L, and Pmaip1, which prevented early acute neuronal cell death and neurological damage (Barteczek et al., 2017). When HIF-1α is overexpressed, development of mitochondrial autophagy is often accompanied by inhibition of the mTOR pathway, thereby increasing neuronal survival, highlighting a novel target molecule that can be used against ischemic neuroprotection (Doeppner et al., 2012; Koh et al., 2015). When mTOR is inactivated by high expression of HIF-1α, AMPK is activated, which may explain the survival of bone marrow mesenchymal stem cells (BMSCs) induced after transplantation of HIF-1α. When BMSCs overexpressing HIF-1α are transplanted into MCAO rats, a reduction in the volume of cerebral infarction, improved neurobehavioral outcomes, inhibited production of pro-inflammatory cytokines, and enhanced secretion of neurotrophic factors occurs, suggesting that HIF-1α may promote BMSCs survival by regulating the activation of AMPK and mTOR to promote autophagy (Lv et al., 2017).

BNIP3 is one of the important target genes of HIF-1α, and BNIP3 gene expression is significantly correlated with HIF-1α gene expression (Feng et al., 2016). Increased expression of HIF-1α promotes BNIP3 gene expression which then activates autophagy. Cerebral ischemia often causes severe mitochondrial damage. By studying the mechanism of mitochondrial autophagy, new targets for ischemic brain damage may be discovered. Along this line of thought, Yuan et al. (2017) found that BNIP3L/NIX is involved in mitochondrial autophagy induced by cerebral ischemia-reperfusion, suggesting that BNIP3L may be a new therapeutic target for ischemic stroke management. In addition, other studies have shown that sirtuin family members have protective effects on neurons and attenuate cerebral ischemia (Carloni et al., 2014; Yang F. et al., 2015; Shimizu et al., 2016).

In addition, post-translational regulation of HIF-1α, SIRT1 and AMPK plays a key role in the control of glycolytic mitochondrial energy axis in response to hypoxic ischemic conditions. Under pseudo hypoxia condition, combination of autophagy reduction, stress, and dysregulation increases the response of the impaired host to hypoxic-ischemic injury (Ham and Raju, 2017). Treatment with dexmedetomidine at the beginning of reperfusion can inhibit autophagy of neurons by up-regulating HIF-1α, thereby protecting the brain from ischemia-reperfusion injury. This finding underscores the potential of this protein as a treatment for acute ischemic injury (Zhang et al., 2016; Zhang and Zhang, 2017; Wang Y.Q. et al., 2018).

### Proteins Associated With Autophagy Formation

#### Beclin1, LC3-II, and P62

Beclin1, LC3-phosphatidylethanolamine conjugates (LC3-II), and P62 are the three major proteins involved in autophagy process. Beclin1 plays an important role in the initiation of autophagy, primarily by forming a trimer with PI3K and Atg14, and continuously recruiting autophagy-associated proteins to mediate the initiation of autophagy (Shao et al., 2016; Qian et al., 2017). The microtubule-associated protein light chain 3 (LC3) undergoes two processing steps, one is proteolytic cleavage of pro-LC3 (LC3 precursor), and the other is delipidization of LC3-PE from autophagosomes. Both processes require the involvement of cysteine protease Atg4. Under the action of Atg4, the LC3 precursor is processed into soluble LC3-I, which is linked to phosphatidyl ethanolamine (PE) under the action of Atg7 and Atg3 to form liposoluble LC3-II-PE, which is involved in autophagocytosis, participating in the extension of the body membrane until autophagic lysosome is formed (Maejima et al., 2013; Khaminets et al., 2016; Chu, 2018). In addition, P62 located in the cytoplasm binds to ubiquitinated proteins, which in turn form a complex with LC3-II protein before it is degraded in lysosomes. During the process of autophagy, P62 is continually consumed (Moscat and Diaz-Meco, 2009; Jiang et al., 2015; Liu W.J. et al., 2016). Therefore, these three proteins are key biomarkers for detecting the level of autophagy. In the case of ischemic stroke, intracellular LC3 content and LC3-I to LC3-II transformation are significantly increased, suggesting high level of autophagy.

Interestingly, a another study showed that transformation level of lncRNA metastasis-associated lung adenocarcinoma transcripts (MALAT1) and autophagy-related proteins LC3-I, LC3-II, and beclin-1 increased after middle cerebral artery occlusion and reperfusion. They found that down-regulation of MALAT1 inhibited beclin-1-dependent autophagy by regulating the expression of miR-30a in cerebral ischemic stroke. MALAT1-miR-30a-Beclin1 was found to form lncRNA-miRNA-mRNA regulation network, thereby reducing neuronal cell death, suggesting that MALAT1 may act as a molecular chaperone of miR-30a that negatively regulates its expression (Wang P. et al., 2014; Guo et al., 2017).

#### Apoptosis-Related Proteins and Heat Shock Protein

Some apoptosis-related genes such as Bcl-2, Bcl-xl, Bax, and caspase can treat stroke by regulating autophagy. Since caspase and Bcl-2 can cleave autophagy-related proteins, a decrease in the level of apoptotic proteins activates autophagy. Previous studies have showed that the GABAβ receptor agonist baclofen can up-regulate the Bcl-2/Bax ratio, increase the activation of Akt, GSK-3β, and ERK, which inhibits autophagy, and significantly alleviate neuronal damage after long-term administration (Liu et al., 2015). This suggests that apoptosis-related genes may attenuate cerebral ischemia by regulating autophagy (Yang Y. et al., 2015; He et al., 2016; Xu et al., 2017).

Heat Shock Protein 27 (Hsp27) has recently become a new effective neuroprotective agent in cerebral ischemia, but the mechanism of Hsp27-mediated neuroprotection is largely unknown. Zhan et al. (2017) found that the expression of phosphorylated MK2 (MAPKAP kinase 2) and Hsp27 were reduced by p38MAPK inhibitor SB203580. Their results showed that inhibition of Hsp27 degradation following autophagy downregulation induced ischemic tolerance after hypoxia post conditioning. It has been suggested that MK2-induced Hsp27 phosphorylation may lead to neuroprotection after hypoxia treatment (Zhan et al., 2017). Blocking the cathepsin-t Bid-mitochondrial apoptosis signaling pathway by inhibiting autophagy and stabilizing the lysosomal membrane is associated with up-regulation of lysosomal Hsp70.1B in astrocytes (Zhou et al., 2017). Further, other studies have found that HSP proteins are involved in the pathophysiology of cerebral ischemia (Qi et al., 2015; Shi et al., 2017; Choi et al., 2018; Yamamoto et al., 2018). This suggests that additional studies on heat shock proteins may provide new options for clinical treatment of stroke.

## Other Related Proteins

Recent studies have shown that α-Synuclein (α-Syn) is a potential therapeutic target for reducing brain damage after stroke. Knock-out of α-Syn significantly reduces infarction in rodent rats with focal cerebral ischemia and promotes neurological recovery. PLK2 (Polo-like kinase 2, the major kinase that mediates α-Syn S129 phosphorylation) knockout in mice during transient focal cerebral ischemia showed better functional recovery and smaller infarcts, indicating a deleterious effect of phosphorylation of the S129 site of α-Syn (Kim et al., 2016).

In the late stage of cerebral ischemia, the expression level of NGF receptor Trk A is decreased, and its endogenous neuroprotective effect is significantly down-regulated. The natural ligand of Trk A, a neurotrophic factor, may then rescue nerve cells by up-regulating the Trk A receptor signaling pathway. Some scholars used glial cell line-derived neurotrophic factor (glial cell line-derived neurotrophic factor, GDNF) and hepatocyte growth factor (HGF) to treat ischemic rats, both of which significantly reduced the infarct size, the number of LC3 and apoptosis-positive cells. These results indicate that GDNF and HGF are not only involved in anti-apoptosis, but are also associated with the inhibition of autophagy (Shang et al., 2010; Yamashita and Abe, 2016). This provides a new scientific basis for the clinical application of neurotrophic factors.

## Prospect

In summary, autophagy and various signal transduction pathways as well as other mechanisms are involved in the development of ischemic stroke. Continuous research and exploration are needed to establish the exact underlying mechanisms. Whether the role of autophagy in ischemic stroke is beneficial or harmful depends not only on the degree of stress in brain cells and mechanism of autophagy, but also on experimental models, detection methods, and research methods (Descloux et al., 2015; Tang et al., 2016; Wang P. et al., 2018; Wolf et al., 2018). Therefore, exploring the occurrence and development of autophagy, strengthening the role of autophagy in different stages of ischemic stroke, studying its molecular mechanisms and signal transduction pathways will help medical practitioners make full use of autophagy in clinical practice, and minimize or avoid the damage caused by autophagy to normal cells in the treatment of ischemic stroke.

## Author Contributions

All authors participated in designing the concept of this manuscript. YS, YZ, and XZ reviewed the literature and drafted the article. XZ, XC, and GY finalized the paper and provided suggestions to improve it.

## Conflict of Interest Statement

The authors declare that the research was conducted in the absence of any commercial or financial relationships that could be construed as a potential conflict of interest.

## References

[B1] Barteczek P. Li L. Ernst A. S. Bohler L. I. Marti H. H. Kunze R. (2017). Neuronal HIF-1alpha and HIF-2alpha deficiency improves neuronal survival and sensorimotor function in the early acute phase after ischemic stroke. *J. Cereb. Blood Flow Metab.* 37 291–306. 10.1177/0271678X15624933 26746864 PMC5363746

[B2] Carloni S. Albertini M. C. Galluzzi L. Buonocore G. Proietti F. Balduini W. (2014). Melatonin reduces endoplasmic reticulum stress and preserves sirtuin 1 expression in neuronal cells of newborn rats after hypoxia-ischemia. *J. Pineal Res.* 57 192–199. 10.1111/jpi.12156 24980917

[B3] Carloni S. Girelli S. Scopa C. Buonocore G. Longini M. Balduini W. (2010). Activation of autophagy and Akt/CREB signaling play an equivalent role in the neuroprotective effect of rapamycin in neonatal hypoxia-ischemia. *Autophagy* 6 366–377. 10.4161/auto.6.3.11261 20168088

[B4] Choi J. I. Ha S. K. Lim D. J. Kim S. D. Kim S. H. (2018). S100ss, Matrix Metalloproteinase-9, D-dimer, and heat shock protein 70 are serologic biomarkers of acute cerebral infarction in a mouse model of transient MCA occlusion. *J. Korean Neurosurg. Soc.* 61 548–558. 10.3340/jkns.2017.0200 29724092 PMC6129755

[B5] Chu C. T. (2018). Mechanisms of selective autophagy and mitophagy: implications for neurodegenerative diseases. *Neurobiol. Dis.* 10.1016/j.nbd.2018.07.015 [Epub ahead of print]. 30030024 PMC6396690

[B6] Cui D. R. Wang L. Jiang W. Qi A. H. Zhou Q. H. Zhang X. L. (2013). Propofol prevents cerebral ischemia-triggered autophagy activation and cell death in the rat hippocampus through the NF-kappaB/p53 signaling pathway. *Neuroscience* 246 117–132. 10.1016/j.neuroscience.2013.04.054 23644056

[B7] Dai S. H. Chen T. Li X. Yue K. Y. Luo P. Yang L. K. (2017). Sirt3 confers protection against neuronal ischemia by inducing autophagy: involvement of the AMPK-mTOR pathway. *Free Radic. Biol. Med.* 108 345–353. 10.1016/j.freeradbiomed.2017.04.005 28396174

[B8] Descloux C. Ginet V. Clarke P. G. Puyal J. Truttmann A. C. (2015). Neuronal death after perinatal cerebral hypoxia-ischemia: focus on autophagy-mediated cell death. *Int. J. Dev. Neurosci.* 45 75–85. 10.1016/j.ijdevneu.2015.06.008 26225751

[B9] Doeppner T. R. Mlynarczuk-Bialy I. Kuckelkorn U. Kaltwasser B. Herz J. Hasan M. R. (2012). The novel proteasome inhibitor BSc2118 protects against cerebral ischaemia through HIF1A accumulation and enhanced angioneurogenesis. *Brain* 135 3282–3297. 10.1093/brain/aws269 23169919

[B10] Doherty J. Baehrecke E. H. (2018). Life, death and autophagy. *Nat. Cell Biol.* 20 1110–1117. 10.1038/s41556-018-0201-5 30224761 PMC9721133

[B11] Feng C. C. Lin C. C. Lai Y. P. Chen T. S. Marthandam Asokan S. Lin J. Y. (2016). Hypoxia suppresses myocardial survival pathway through HIF-1alpha-IGFBP-3-dependent signaling and enhances cardiomyocyte autophagic and apoptotic effects mainly via FoxO3a-induced BNIP3 expression. *Growth Factors* 34 73–86. 10.1080/08977194.2016.1191480 27366871

[B12] Ferrer I. Friguls B. Dalfo E. Planas A. M. (2003). Early modifications in the expression of mitogen-activated protein kinase (MAPK/ERK), stress-activated kinases SAPK/JNK and p38, and their phosphorylated substrates following focal cerebral ischemia. *Acta Neuropathol.* 105 425–437. 12677442 10.1007/s00401-002-0661-2

[B13] Guo D. Ma J. Yan L. Li T. Li Z. Han X. (2017). Down-regulation of lncrna MALAT1 attenuates neuronal cell death through suppressing beclin1-dependent autophagy by regulating Mir-30a in cerebral ischemic stroke. *Cell Physiol. Biochem.* 43 182–194. 10.1159/000480337 28854438

[B14] Halestrap A. P. (2006). Calcium, mitochondria and reperfusion injury: a pore way to die. *Biochem. Soc. Trans.* 34 232–237. 10.1042/BST0340232 16545083

[B15] Ham P. B. III Raju R. (2017). Mitochondrial function in hypoxic ischemic injury and influence of aging. *Prog. Neurobiol.* 157 92–116. 10.1016/j.pneurobio.2016.06.006 27321753 PMC5161736

[B16] Han D. Scott E. L. Dong Y. Raz L. Wang R. Zhang Q. (2015). Attenuation of mitochondrial and nuclear p38alpha signaling: a novel mechanism of estrogen neuroprotection in cerebral ischemia. *Mol. Cell. Endocrinol.* 400 21–31. 10.1016/j.mce.2014.11.010 25462588

[B17] He G. Xu W. Tong L. Li S. Su S. Tan X. (2016). Gadd45b prevents autophagy and apoptosis against rat cerebral neuron oxygen-glucose deprivation/reperfusion injury. *Apoptosis* 21 390–403. 10.1007/s10495-016-1213-x 26882903

[B18] Hei C. Liu P. Yang X. Niu J. Li P. A. (2017). Inhibition of mTOR signaling confers protection against cerebral ischemic injury in acute hyperglycemic rats. *Int. J. Biol. Sci.* 13 878–887. 10.7150/ijbs.18976 28808420 PMC5555105

[B19] Hong Y. Shao A. Wang J. Chen S. Wu H. McBride D. W. (2014). Neuroprotective effect of hydrogen-rich saline against neurologic damage and apoptosis in early brain injury following subarachnoid hemorrhage: possible role of the Akt/GSK3beta signaling pathway. *PLoS One* 9:e96212. 10.1371/journal.pone.0096212 24763696 PMC3999200

[B20] Huang L. Chen C. Zhang X. Li X. Chen Z. Yang C. (2018). Neuroprotective effect of curcumin against cerebral ischemia-reperfusion via mediating autophagy and inflammation. *J. Mol. Neurosci.* 64 129–139. 10.1007/s12031-017-1006-x 29243061

[B21] Jiang M. Li J. Peng Q. Liu Y. Liu W. Luo C. (2014). Neuroprotective effects of bilobalide on cerebral ischemia and reperfusion injury are associated with inhibition of pro-inflammatory mediator production and down-regulation of JNK1/2 and p38 MAPK activation. *J. Neuroinflammation* 11:167. 10.1186/s12974-014-0167-6 25256700 PMC4189683

[B22] Jiang T. Harder B. Rojo de la Vega M. Wong P. K. Chapman E. Zhang D. D. (2015). p62 links autophagy and Nrf2 signaling. *Free Radic. Biol. Med.* 88 199–204. 10.1016/j.freeradbiomed.2015.06.014 26117325 PMC4628872

[B23] Khaminets A. Behl C. Dikic I. (2016). Ubiquitin-dependent and independent signals in selective autophagy. *Trends Cell Biol.* 26 6–16. 10.1016/j.tcb.2015.08.010 26437584

[B24] Kim T. Mehta S. L. Kaimal B. Lyons K. Dempsey R. J. Vemuganti R. (2016). Poststroke induction of alpha-synuclein mediates ischemic brain damage. *J. Neurosci.* 36 7055–7065. 10.1523/JNEUROSCI.1241-16.2016 27358461 PMC4994709

[B25] Koh H. S. Chang C. Y. Jeon S. B. Yoon H. J. Ahn Y. H. Kim H. S. (2015). The HIF-1/glial TIM-3 axis controls inflammation-associated brain damage under hypoxia. *Nat Commun.* 6:6340. 10.1038/ncomms7340 25790768 PMC4383004

[B26] Kuma A. Hatano M. Matsui M. Yamamoto A. Nakaya H. Yoshimori T. (2004). The role of autophagy during the early neonatal starvation period. *Nature* 432 1032–1036. 10.1038/nature03029 15525940

[B27] Li H. Wu J. Shen H. Yao X. Liu C. Pianta S. (2018). Autophagy in hemorrhagic stroke: mechanisms and clinical implications. *Prog. Neurobiol.* 16 79–97. 10.1016/j.pneurobio.2017.04.002 28414101

[B28] Li H. Zhou S. Wu L. Liu K. Zhang Y. Ma G. (2015). The role of p38MAPK signal pathway in the neuroprotective mechanism of limb postconditioning against rat cerebral ischemia/reperfusion injury. *J. Neurol. Sci.* 357 270–275. 10.1016/j.jns.2015.08.004 26282496

[B29] Li W. L. Yu S. P. Chen D. Yu S. S. Jiang Y. J. Genetta T. (2013). The regulatory role of NF-kappaB in autophagy-like cell death after focal cerebral ischemia in mice. *Neuroscience* 244 16–30. 10.1016/j.neuroscience.2013.03.045 23558089 PMC3916093

[B30] Liu H. Zhang Y. Wu H. D’Alessandro A. Yegutkin G. G. Song A. (2016). Beneficial role of erythrocyte adenosine A2B receptor-mediated AMP-activated protein kinase activation in high-altitude hypoxia. *Circulation* 134 405–421. 10.1161/CIRCULATIONAHA.116.021311 27482003 PMC6168195

[B31] Liu L. Li C. J. Lu Y. Zong X. G. Luo C. Sun J. (2015). Baclofen mediates neuroprotection on hippocampal CA1 pyramidal cells through the regulation of autophagy under chronic cerebral hypoperfusion. *Sci. Rep.* 5:14474. 10.1038/srep14474 26412641 PMC4585985

[B32] Liu W. J. Ye L. Huang W. F. Guo L. J. Xu Z. G. Wu H. L. (2016). p62 links the autophagy pathway and the ubiqutin-proteasome system upon ubiquitinated protein degradation. *Cell Mol. Biol. Lett.* 21:29. 10.1186/s11658-016-0031-z 28536631 PMC5415757

[B33] Liu Y. Xue X. Zhang H. Che X. Luo J. Wang P. (2018). Neuronal-targeted TFEB rescues dysfunction of the autophagy-lysosomal pathway and alleviates ischemic injury in permanent cerebral ischemia. *Autophagy* 2018 1–17. 10.1080/15548627.2018.1531196 30304977 PMC6351122

[B34] Lv B. Li F. Han J. Fang J. Xu L. Sun C. (2017). Hif-1alpha overexpression improves transplanted bone mesenchymal stem cells survival in Rat MCAO stroke model. *Front. Mol. Neurosci.* 10:80. 10.3389/fnmol.2017.00080 28424584 PMC5372780

[B35] Maejima Y. Kyoi S. Zhai P. Liu T. Li H. Ivessa A. (2013). Mst1 inhibits autophagy by promoting the interaction between Beclin1 and Bcl-2. *Nat. Med.* 19 1478–1488. 10.1038/nm.3322 24141421 PMC3823824

[B36] Morselli E. Tasdemir E. Maiuri M. C. Galluzzi L. Kepp O. Criollo A. (2008). Mutant p53 protein localized in the cytoplasm inhibits autophagy. *Cell Cycle* 7 3056–3061. 10.4161/cc.7.19.6751 18818522

[B37] Moscat J. Diaz-Meco M. T. (2009). p62 at the crossroads of autophagy, apoptosis, and cancer. *Cell* 137 1001–1004. 10.1016/j.cell.2009.05.023 19524504 PMC3971861

[B38] Nikoletopoulou V. Papandreou M. E. Tavernarakis N. (2015). Autophagy in the physiology and pathology of the central nervous system. *Cell Death Differ.* 22 398–407. 10.1038/cdd.2014.204 25526091 PMC4326580

[B39] Nitatori T. Sato N. Waguri S. Karasawa Y. Araki H. Shibanai K. (1995). Delayed neuronal death in the CA1 pyramidal cell layer of the gerbil hippocampus following transient ischemia is apoptosis. *J. Neurosci.* 15 1001–1011. 10.1523/JNEUROSCI.15-02-01001.1995 7869078 PMC6577848

[B40] Qi J. Liu Y. Yang P. Chen T. Liu X. Z. Yin Y. (2015). Heat shock protein 90 inhibition by 17-Dimethylaminoethylamino-17-demethoxygeldanamycin protects blood-brain barrier integrity in cerebral ischemic stroke. *Am. J. Transl. Res.* 7 1826–1837. 26692927 PMC4656760

[B41] Qian X. Li X. Cai Q. Zhang C. Yu Q. Jiang Y. (2017). Phosphoglycerate kinase 1 phosphorylates beclin1 to induce autophagy. *Mol. Cell.* 65 917.e6–931.e6. 10.1016/j.molcel.2017.01.027 28238651 PMC5389741

[B42] Rami A. Kogel D. (2008). Apoptosis meets autophagy-like cell death in the ischemic penumbra: two sides of the same coin? *Autophagy* 4 422–426. 18319639 10.4161/auto.5778

[B43] Ronnett G. V. Ramamurthy S. Kleman A. M. Landree L. E. Aja S. (2009). AMPK in the brain: its roles in energy balance and neuroprotection. *J. Neurochem.* 109(Suppl. 1), 17–23. 10.1111/j.1471-4159.2009.05916.x 19393004 PMC2925428

[B44] Sciarretta S. Forte M. Frati G. Sadoshima J. (2018). New insights into the role of mtor signaling in the cardiovascular system. *Circ. Res.* 122 489–505. 10.1161/CIRCRESAHA.117.311147 29420210 PMC6398933

[B45] Shang J. Deguchi K. Yamashita T. Ohta Y. Zhang H. Morimoto N. (2010). Antiapoptotic and antiautophagic effects of glial cell line-derived neurotrophic factor and hepatocyte growth factor after transient middle cerebral artery occlusion in rats. *J. Neurosci. Res.* 88 2197–2206. 10.1002/jnr.22373 20175208

[B46] Shao A. Wang Z. Wu H. Do-ng X. Li Y. Tu S. (2016). Enhancement of autophagy by histone deacetylase inhibitor trichostatin a ameliorates neuronal apoptosis after subarachnoid hemorrhage in rats. *Mol. Neurobiol.* 53 18–27. 10.1007/s12035-014-8986-0 25399954

[B47] Shi Y. Jiang X. Zhang L. Pu H. Hu X. Zhang W. (2017). Endothelium-targeted overexpression of heat shock protein 27 ameliorates blood-brain barrier disruption after ischemic brain injury. *Proc. Natl. Acad. Sci. U.S.A.* 114 E1243–E1252. 10.1073/pnas.1621174114 28137866 PMC5320958

[B48] Shimizu K. Quillinan N. Orfila J. E. Herson P. S. (2016). Sirtuin-2 mediates male specific neuronal injury following experimental cardiac arrest through activation of TRPM2 ion channels. *Exp. Neurol.* 275(Pt 1), 78–83. 10.1016/j.expneurol.2015.10.014 26522013 PMC5193101

[B49] Shioda N. Han F. Fukunaga K. (2009). Role of Akt and ERK signaling in the neurogenesis following brain ischemia. *Int. Rev. Neurobiol.* 85 375–387. 10.1016/S0074-7742(09)85026-5 19607982

[B50] Song Y. Q. Zou H. L. Zhao Y. J. Yu L. Q. Tan Z. X. Kong R. (2016). Activation of p38-mitogen-activated protein kinase contributes to ischemia reperfusion in rat brain. *Genet. Mol. Res.* 15 1–13. 10.4238/gmr.15038492 27706770

[B51] Tang Y. C. Tian H. X. Yi T. Chen H. B. (2016). The critical roles of mitophagy in cerebral ischemia. *Protein Cell* 7 699–713. 10.1007/s13238-016-0307-0 27554669 PMC5055489

[B52] Vercelli A. Biggi S. Sclip A. Repetto I. E. Cimini S. Falleroni F. (2015). Exploring the role of MKK7 in excitotoxicity and cerebral ischemia: a novel pharmacological strategy against brain injury. *Cell Death Dis.* 6:e1854. 10.1038/cddis.2015.226 26270349 PMC4558515

[B53] Wang J. F. Mei Z. G. Fu Y. Yang S. B. Zhang S. Z. Huang W. F. (2018). Puerarin protects rat brain against ischemia/reperfusion injury by suppressing autophagy via the AMPK-mTOR-ULK1 signaling pathway. *Neural Regen. Res.* 13 989–998. 10.4103/1673-5374.233441 29926825 PMC6022469

[B54] Wang P. Liang J. Li Y. Li J. Yang X. Zhang X. (2014). Down-regulation of miRNA-30a alleviates cerebral ischemic injury through enhancing beclin 1-mediated autophagy. *Neurochem. Res.* 39 1279–1291. 10.1007/s11064-014-1310-6 24771295

[B55] Wang P. Shao B. Z. Deng Z. Chen S. Yue Z. Miao C. Y. (2018). Autophagy in ischemic stroke. *Prog. Neurobiol.* 163-164 98–117. 10.1016/j.pneurobio.2018.01.001 29331396

[B56] Wang Y. Zhen Y. Wu X. Jiang Q. Li X. Chen Z. (2015). Vitexin protects brain against ischemia/reperfusion injury via modulating mitogen-activated protein kinase and apoptosis signaling in mice. *Phytomedicine* 22 379–384. 10.1016/j.phymed.2015.01.009 25837275

[B57] Wang Y. Q. Tang Y. F. Yang M. K. Huang X. Z. (2018). Dexmedetomidine alleviates cerebral ischemia-reperfusion injury in rats via inhibition of hypoxia-inducible factor-1α. *J. Cell Biochem.* 10.1002/jcb.28058 [Epub ahead of print]. 30456861

[B58] Wolf M. S. Bayir H. Kochanek P. M. Clark R. S. B. (2018). The role of autophagy in acute brain injury: a state of flux? *Neurobiol. Dis.* 10.1016/j.nbd.2018.04.018 [Epub ahead of print]. 29704549 PMC6203674

[B59] Xu J. Huai Y. Meng N. Dong Y. Liu Z. Qi Q. (2017). L-3-n-butylphthalide activates Akt/mTOR signaling, inhibits neuronal apoptosis and autophagy and improves cognitive impairment in mice with repeated cerebral ischemia-reperfusion injury. *Neurochem. Res.* 42 2968–2981. 10.1007/s11064-017-2328-3 28620824

[B60] Xu W. Gao L. Li T. Zheng J. Shao A. Zhang J. (2018). Mesencephalic astrocyte-derived neurotrophic factor (MANF) protects against neuronal apoptosis via activation of Akt/MDM2/p53 signaling pathway in a rat model of intracerebral hemorrhage. *Front. Mol. Neurosci.* 11:176. 10.3389/fnmol.2018.00176 29896089 PMC5987019

[B61] Xue L. X. Xu Z. H. Wang J. Q. Cui Y. Liu H. Y. Liang W. Z. (2016). Activin A/Smads signaling pathway negatively regulates oxygen glucose deprivation-induced autophagy via suppression of JNK and p38 MAPK pathways in neuronal PC12 cells. *Biochem. Biophys. Res. Commun.* 480 355–361. 10.1016/j.bbrc.2016.10.050 27769861

[B62] Yamamoto Y. Hosoda K. Imahori T. Tanaka J. Matsuo K. Nakai T. (2018). Pentose phosphate pathway activation via HSP27 phosphorylation by ATM kinase: a putative endogenous antioxidant defense mechanism during cerebral ischemia-reperfusion. *Brain Res.* 1687 82–94. 10.1016/j.brainres.2018.03.001 29510140

[B63] Yamashita T. Abe K. (2016). Recent progress in therapeutic strategies for ischemic stroke. *Cell Transplant.* 25 893–898. 10.3727/096368916X690548 26786838

[B64] Yang F. Zhou L. Wang D. Wang Z. Huang Q. Y. (2015). Minocycline ameliorates hypoxia-induced blood-brain barrier damage by inhibition of HIF-1alpha through SIRT-3/PHD-2 degradation pathway. *Neuroscience* 304 250–259. 10.1016/j.neuroscience.2015.07.051 26211444

[B65] Yang Y. Gao K. Hu Z. Li W. Davies H. Ling S. (2015). Autophagy upregulation and apoptosis downregulation in DAHP and triptolide treated cerebral ischemia. *Mediators Inflamm.* 2015 120198. 10.1155/2015/120198 25729215 PMC4333273

[B66] Yu J. Li C. Ding Q. Que J. Liu K. Wang H. (2017). Netrin-1 ameliorates blood-brain barrier impairment secondary to ischemic stroke via the activation of PI3K pathway. *Front. Neurosci.* 11:700. 10.3389/fnins.2017.00700 29311781 PMC5732993

[B67] Yuan Y. Zheng Y. Zhang X. Chen Y. Wu X. Wu J. (2017). BNIP3L/NIX-mediated mitophagy protects against ischemic brain injury independent of PARK2. *Autophagy* 13 1754–1766. 10.1080/15548627.2017.1357792 28820284 PMC5640199

[B68] Zhan L. Liu L. Li K. Wu B. Liu D. Liang D. (2017). Neuroprotection of hypoxic postconditioning against global cerebral ischemia through influencing posttranslational regulations of heat shock protein 27 in adult rats. *Brain Pathol.* 27 822–838. 10.1111/bpa.12472 27936516 PMC8029298

[B69] Zhang W. Zhang J. (2017). Dexmedetomidine preconditioning protects against lung injury induced by ischemia-reperfusion through inhibition of autophagy. *Exp. Ther. Med.* 14 973–980. 10.3892/etm.2017.4623 28810549 PMC5526121

[B70] Zhang W. Zhang J. Q. Meng F. M. Xue F. S. (2016). Dexmedetomidine protects against lung ischemia-reperfusion injury by the PI3K/Akt/HIF-1alpha signaling pathway. *J. Anesth.* 30 826–833. 10.1007/s00540-016-2214-1 27412350

[B71] Zhang Y. Miao J. M. (2018). Ginkgolide K promotes astrocyte proliferation and migration after oxygen-glucose deprivation via inducing protective autophagy through the AMPK/mTOR/ULK1 signaling pathway. *Eur. J. Pharmacol* 832 96–103. 10.1016/j.ejphar.2018.05.029 29787772

[B72] Zhou X. Y. Luo Y. Zhu Y. M. Liu Z. H. Kent T. A. Rong J. G. (2017). Inhibition of autophagy blocks cathepsins-tBid-mitochondrial apoptotic signaling pathway via stabilization of lysosomal membrane in ischemic astrocytes. *Cell Death Dis.* 8:e2618. 10.1038/cddis.2017.34 28206988 PMC5386481

